# Retinal ganglion cells adapt to ionic stress in experimental glaucoma

**DOI:** 10.3389/fnins.2023.1142668

**Published:** 2023-03-27

**Authors:** Andrew M. Boal, Nolan R. McGrady, Joseph M. Holden, Michael L. Risner, David J. Calkins

**Affiliations:** Department of Ophthalmology and Visual Sciences, Vanderbilt Eye Institute, Vanderbilt University Medical Center, Nashville, TN, United States

**Keywords:** neurodegeneration, retinal ganglion cells, glaucoma, excitability, potassium, physiology, action potential

## Abstract

**Introduction:**

Identification of early adaptive and maladaptive neuronal stress responses is an important step in developing targeted neuroprotective therapies for degenerative disease. In glaucoma, retinal ganglion cells (RGCs) and their axons undergo progressive degeneration resulting from stress driven by sensitivity to intraocular pressure (IOP). Despite therapies that can effectively manage IOP many patients progress to vision loss, necessitating development of neuronal-based therapies. Evidence from experimental models of glaucoma indicates that early in the disease RGCs experience altered excitability and are challenged with dysregulated potassium (K^+^) homeostasis. Previously we demonstrated that certain RGC types have distinct excitability profiles and thresholds for depolarization block, which are associated with sensitivity to extracellular K^+^.

**Methods:**

Here, we used our inducible mouse model of glaucoma to investigate how RGC sensitivity to K^+^ changes with exposure to elevated IOP.

**Results:**

In controls, conditions of increased K^+^ enhanced membrane depolarization, reduced action potential generation, and widened action potentials. Consistent with our previous work, 4 weeks of IOP elevation diminished RGC light-and current-evoked responses. Compared to controls, we found that IOP elevation reduced the effects of increased K^+^ on depolarization block threshold, with IOP-exposed cells maintaining greater excitability. Finally, IOP elevation did not alter axon initial segment dimensions, suggesting that structural plasticity alone cannot explain decreased K^+^ sensitivity.

**Discussion:**

Thus, in response to prolonged IOP elevation RGCs undergo an adaptive process that reduces sensitivity to changes in K^+^ while diminishing excitability. These experiments give insight into the RGC response to IOP stress and lay the groundwork for mechanistic investigation into targets for neuroprotective therapy.

## 1. Introduction

Glaucoma is the leading cause of irreversible vison loss worldwide (Tham et al., 2014). The disease involves progressive degeneration of retinal ganglion cells (RGCs) and their axons, which carry visual information from the eye to central targets in the brain. Aging is the leading risk factor, though sensitivity to intraocular pressure (IOP) is the only modifiable risk factor. In glaucoma, stress evolving from sensitivity to IOP challenges RGC axons as they pass unmyelinated through the optic nerve head of the retina. Many patients continue to lose vision despite efforts to manage IOP with topical and surgical hypotensive therapies (Heijl et al., 2002), underscoring the need to identify new therapeutics based on mechanistic understanding of how RGCs and their axons respond to glaucomatous stress.

Development of neuronal-based therapies for the treatment of glaucoma requires identification of targets involved in pathophysiology, target-specific therapeutics, and biomarkers to assay outcomes (Calkins, 2021). Early progression in experimental glaucoma involves enhanced RGC excitability with a concurrent reduction in axon function (Risner et al., 2018). Prolonged stress ultimately overcomes the adaptive mechanisms and leads to RGC degeneration (Sappington et al., 2010; Naguib et al., 2021; Risner et al., 2021, 2022). The RGC population is heterogeneous (Sanes and Masland, 2015; Baden et al., 2016; Bae et al., 2018; Tran et al., 2019), with RGC-intrinsic factors shaping their individual response properties (Emanuel et al., 2017; Werginz et al., 2020; Wienbar and Schwartz, 2022). Importantly, such intrinsic differences may predispose certain RGC types to be particularly sensitive to IOP-related stress (Della Santina et al., 2013; El-Danaf and Huberman, 2015; Ou et al., 2016; Risner et al., 2021). Previously, we established that different RGC subtypes exhibit varied sensitivities to elevated extracellular potassium (Boal et al., 2022). Dysregulation of potassium ion (K^+^) homeostasis and channel expression contribute to altered excitability in neurodegenerative diseases, including glaucoma, and represent potential targets for early diagnosis and treatment (Hall et al., 2015; Frazzini et al., 2016; Cacace et al., 2019; Fischer et al., 2019a,b; Kim et al., 2021).

Here, we utilized our inducible mouse model of glaucoma (Sappington et al., 2010; Calkins et al., 2018) to investigate how prolonged exposure to elevated IOP changes RGC sensitivity to acutely elevated extracellular K^+^. Following 4 weeks of IOP elevation, alpha ON-sustained (αON-S) and alpha OFF-sustained (αOFF-S) RGCs had reduced responses to light and depolarizing current stimulation, consistent with previous results (Risner et al., 2021, 2022). In controls with normal IOP, challenging RGCs with high extracellular K^+^ led to membrane depolarization, blunted spike rate, and action potential (AP) widening in both αON-S and αOFF-S cells. IOP elevation reduced the effects of elevated K^+^ in both RGC types. Compared to controls, RGCs exposed to elevated IOP were less depolarized and maintained greater current-evoked spiking during acute K^+^ elevation. Furthermore, K^+^-dependent AP widening was decreased, though the impact of IOP on AP widths differed for αON-S and αOFF-S cells. Immunolabeling of the axon initial segment (AIS), the site of AP initiation in neurons, revealed that IOP elevation did not structurally alter AIS scaffolding for either RGC type.

These results suggest that, after 4 weeks of IOP elevation, RGCs undergo an adaptive process that reduces sensitivity to acutely elevated K^+^ while diminishing their excitability. Differences between αON-S and αOFF-S in how AP widths vary with IOP exposure and K^+^ conditions support evidence for cell-type specific responses to stress. This adaptation involves altered AP generation, indicating an axogenic process, but it is not solely reflective of axonal structural plasticity.

## 2. Materials and methods

### 2.1. Animals

We obtained 15 C57Bl6/J mice (8 males, 7 females, 12–20 weeks old) from Jackson Laboratories (Bar Harbor, ME). These numbers were determined, based upon our previous experience with this model and recording strategy (Risner et al., 2018, 2021; Boal et al., 2022), to provide a sufficient number of each cell type for statistical comparisons. Mice were housed at the Vanderbilt University Division of Animal Care and maintained on 12-h light/dark cycle. Animals were allowed water and standard rodent chow *ad libitum*. All animal experiments were reviewed and approved by the Vanderbilt University Medical Center Institutional Animal Care and Use Committee.

### 2.2. Intraocular pressure elevation and measurement

Mice were anesthetized with isoflurane (2.5%) and administered tropicamide (1%), proparacaine (0.5%), and lubricating drops in both eyes. For the 4 week intraocular pressure (IOP) elevation group (4wk IOP) we bilaterally injected 1.5 μL of 15 μm polystyrene microbeads (Invitrogen, Carlsbad, CA) into the anterior chamber of the eye (Sappington et al., 2010) using borosilicate glass pipette attached to a micromanipulator (M3301R, WPI, Sarasota, FL), driven by a microsyringe pump (DMP, WPI, Sarasota, FL). For the 4wk saline control group (4wk Ctrl) we bilaterally injected an equal volume of sterile phosphate-buffered saline (PBS) into the anterior chamber using the same system. Mice were injected in cohorts of five at a time. For 4wk Ctrl, a second cohort of five was done because an insufficient number of cells of interest were recorded from the first. Animals of both sexes were evenly split between experimental groups (*p* = 0.5581, Chi-squared test).

For IOP measurements, mice were lightly anesthetized with isoflurane (2%) and pressures were measured using rebound tonometry (iCare Tonolab; Vantaa, Finland). IOP for each eye was determined as the mean of 15 consecutive measurements. For the 2 days preceding anterior chamber injections we measured IOP for each group and averaged these values to determine baseline IOP. Beginning 2 days post-injection, IOP was measured three times per week for the duration of the 4 week experimental period.

### 2.3. Electrophysiology

Approximately 4 weeks (±2 days) following anterior chamber injection mice were euthanized *via* cervical dislocation and decapitation, eyes were enucleated, and the retinas were dissected out under long-wavelength illumination (630 nm, 800 μW/cm^2^, FND/FG, Ushio, Cypress, CA). Retinas were placed in carbogen-saturated Ames’ medium (US Biologic, Memphis, TN) supplemented with 20 mM D-glucose and 22.6 mM NaHCO_3_ (pH 7.4, 290 Osm). Each retina was mounted flat onto a physiological chamber, inner retina facing upwards, and perfused at a rate of 2 mL/min with Ames’ medium maintained at 35°C (Model TC-344C, Warner Instruments, Hamden, CT).

Retinal ganglion cells (RGCs) were viewed under differential interference contrast (DIC) using an Andor CCD camera attached to an Olympus BX50 upright microscope at 40x magnification. RGCs with large somas were targeted for intracellular recording with pipettes pulled from borosilicate glass (I.D. 0.86 mm, O.D. 1.5 mm; Sutter Instruments, Novato, CA) and filled with (in mM): 125 K-gluconate, 10 KCl, 10 HEPES, 10 EGTA, 4 Mg-ATP, 1 Na-GTP, and 0.1 ALEXA 555 dye (Invitrogen, Carlsbad, CA). The intracellular solution pH was 7.35 and osmolarity was 285 Osm. Recording pipettes filled with intracellular solution had a resistance between 4 and 8 MΩ. Whole-cell current-clamp signals were amplified (Multiclamp 700B, Molecular Devices, San Jose, CA) and digitized at 10 kHz (Digidata 1550A, Molecular Devices, San Jose, CA). Access resistance was monitored periodically during recordings and maintained ≤ 30 MΩ.

We measured resting membrane potential (RMP), spontaneous spiking, light-evoked spike activity (full-field 365 + 460 nm, 3.4 mW/cm^2^, 3-s duration, CoolLED, pE-4,000, Andover, United Kingdom), and current-evoked spiking while clamping the cell at 0 pA. Current-evoked spiking was measured during stepwise application of 1 s depolarizing current pulses, ranging from 0 to +300 pA in 10 pA increments, with a 2 s inter-stimulus interval. Current was clamped at 0 pA between pulses.

### 2.4. High extracellular potassium recordings

A second batch of Ames’ medium was prepared as described above, but with an additional 5 mM of KCl (i.e., high K^+^), bringing the total K^+^ concentration to 8 mM. Following completion of baseline recordings, high K^+^ medium was washed into the recording chamber while RGC membrane voltage was continuously recorded. Prior to high K^+^ experimental recordings, we allowed a wash on period of 5–6 min, allowing RGC membrane potential to stabilize. Recordings (RMP, spontaneous activity, light-evoked spiking, current-evoked spiking) were then performed as described above. After high K^+^ experiments the extracellular medium was switched back to the baseline solution and RGC membrane voltage was continuously measured during a wash off period of 10–20 min, allowing RGCs to recover baseline RMP and spontaneous activity. Full recovery typically took 10–15 min, although it occasionally required up to 20 min. We limited the number of experimental protocols to reduce the time of high K^+^ exposure. Furthermore, to limit potential cumulative effects of K^+^ wash on/off, we limited the total number of cells that were recorded under high K^+^ to no more than 3 from each retina.

### 2.5. RGC physiology analysis

Raw data files from electrophysiologic recordings were analyzed in Python 3.9 using the pyABF 2.3.5 (Harden, 2022) and SciPy 1.7.1 modules (Virtanen et al., 2020). Action potentials (APs) were detected from membrane voltage data using the SciPy “find_peaks” function with parameters of 20 mV minimum prominence and a distance threshold of 1.5 ms. Spike rates for current-evoked spiking protocols were reported as the average rate for 2 adjacent 10 pA increments of stimulation (20 pA bins). For AP width measurements, a cubic spline function was fit to each AP waveform and half-width was measured as the duration, in ms, where the membrane potential was above the midway point between AP peak and minimum after-hyperpolarization.

### 2.6. Immunohistochemistry and imaging

Immediately following recordings, retinas were fixed in 4% paraformaldehyde and incubated at 4°C for 24 h. After fixation, retinas were immunolabeled for choline acetyltransferase (ChAT, 1:100; Millipore, Burlington, MA, Cat. #AB144P) and ankyrin-G (AnkG, 1:200; NeuroMab N106/36; Antibodies, Inc. Cat. # 75-146). Tissue was blocked in 5% normal donkey serum for 2 h, then incubated in primary antibodies for 3 d at 4°C, and finally incubated for 2 h at room temperature with donkey anti-goat Alexa 405 and donkey anti-mouse Alexa 488 secondary antibodies (Jackson ImmunoResearch, West Grove, PA). Z-stack images of dye-filled RGCs were obtained using an Olympus FV1000 inverted microscope at 40x magnification. Image analysis, including creating orthogonal projections used for visualization of dendritic stratification depth, was performed using ImageJ (NIH, Bethesda, MD).

### 2.7. Axon initial segment analysis

We evaluated 11 4wk IOP cells (5 αON-S and 6 αOFF-S) and 18 4wk Ctrl cells (9 αON-S and 9 αOFF-S) with identifiable axon initial segments (AISs) as defined by a segment of ankyrin-G (AnkG) labeling that colocalized to a filled RGC axon. AnkG fluorescence was measured in ImageJ starting from the edge of the soma along the axon in a max-intensity Z projection limited to the extent of the axonal process. Background fluorescence was subtracted from AnkG intensity profiles using a rolling ball filter with a radius equal to approximately 15% of the data length. Smoothed AnkG profiles were generated using a Savitzky–Golay filter with a first order polynomial fit. Axon initial segment (AIS) bounds were algorithmically defined as the extent where smoothed AnkG values were greater than 50% of the difference between baseline and maximum intensity.

### 2.8. Data analysis and statistical tests

All data are reported as mean ± standard error of the mean (SEM) unless otherwise indicated. All statistical tests were performed in GraphPad Prism 9 (Graphpad Software, San Diego, CA). All data sets were checked for normality. Where appropriate, parametric statistical tests (unpaired *t*-test, paired *t*-test, 2-way ANOVA, simple linear regression) were performed. When data were not normally distributed, appropriate nonparametric tests (e.g., Mann–Whitney test) were performed. For ANOVA tests, *p*-values were corrected for multiple comparisons. Where noted, determination of the influence of sex on high K^+^ -induced change in RMP was determined by multiple linear regression modeling (∆RMP ~ Intercept + Cell Type + Experimental Group + Sex). We defined statistical significance as a *p*-value of 0.05 or less. Exact *p*-values and the specific statistical test used for each analysis are listed in the figure legends or results text.

## 3. Results

### 3.1. Elevated IOP alters RGC electrophysiology

**Figure 1:**
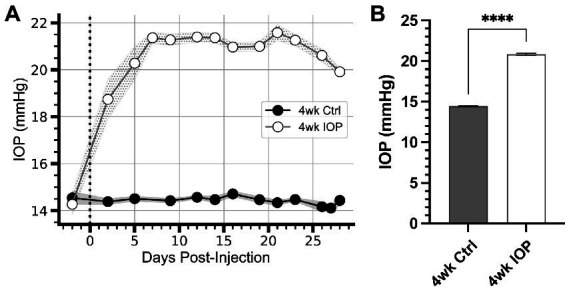
IOP elevation due to microbead occlusion of the anterior chamber. **(A)** Intraocular pressure (IOP) of microbead-injected eyes (4wk IOP) increases rapidly following injection (vertical dotted line) and remain elevated for the duration of the 4 weeks. The IOP of saline-injected eyes (4wk Ctrl) remains unchanged from baseline. Shaded region: ± standard error of the mean. **(B)** Mean IOP for each eye across all days following microbead injection significantly elevates compared to Ctrl (*p* = 0.000046, unpaired *t*-test). Error bars: ± standard error of the mean. ****p < 0.0001.

We performed bilateral injections of either polystyrene microbeads to occlude the anterior chamber (*n* = 5 animals, 10 eyes) or sterile phosphate-buffered saline (*n* = 10 animals, 20 eyes) and measured intraocular pressure (IOP) for 4 weeks (Figure 1). Following injections, IOP in saline-injected eyes remained unchanged from baseline (Figure 1A). Following microbead occlusion, IOP increased 46% above their baseline (Figure 1A), exceeding IOP in saline control eyes by 44% (Figure 1B, *p* < 0.0001). IOP elevation in microbead eyes was sustained for the duration of the experiment (Figure 1A).

**Figure 2:**
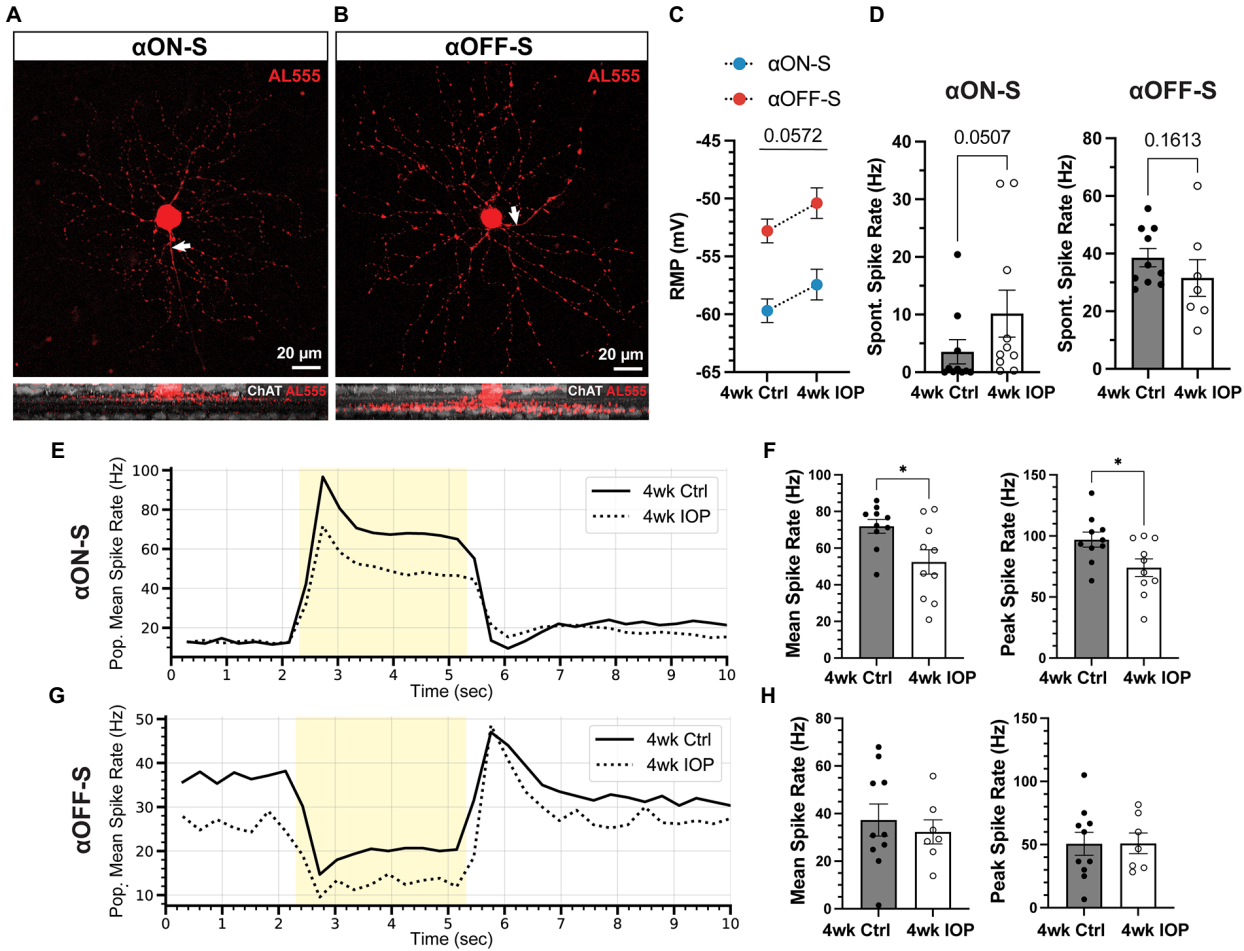
Elevated IOP alters membrane and light-evoked spiking characteristics in αON-S and αOFF-S RGCs. **(A,B)** Morphologic and physiologic characterization of retinal ganglion cells (RGCs). Patched cells were filled with Alexa-fluor 555 dye (AL555, red) and morphologically reconstructed with confocal microscopy. Representative maximum intensity projections of alpha ON-sustained (αON-S, **A**) and alpha OFF-sustained (αOFF-S, **B**) RGCs demonstrate characteristic soma size and dendritic branching patterns (upper). White arrows indicate the axonal projection. Orthogonal projections of representative AL555-filled cells co-labeled for choline acetyltransferase (ChAT, white) demonstrate the branching of αON-S and αOFF-S dendrites in the ON- and OFF-sublaminas of the inner plexiform layer, respectively (lower). **(C)** Resting membrane potentials (RMP) for both cell types from 4wk Ctrl and IOP groups. RGC RMPs in the 4wk IOP group are more depolarized than controls (*p* = 0.0572, 2-way ANOVA). **(D)** Spontaneous spiking rates for αON-S and αOFF-S RGCs. αON-S cells in the 4wk IOP group trend toward greater spontaneous spiking (*p* = 0.0507, Mann–Whitney test), whereas 4wk IOP αOFF-S cells trend toward less spontaneous spiking (*p* = 0.1613, Mann–Whitney test). **(E)** Mean firing rates of αON-S cells in the 4wk Ctrl and 4wk IOP groups binned into 200 ms intervals during light stimulation (yellow). **(F)** Mean (left) and peak (right) light-evoked spike rates for αON-S cells. 4wk IOP decreases both measures (mean: *p* = 0.0202, unpaired *t*-test; peak: *p* = 0.0251, unpaired *t*-test). **(G)** Mean firing rates of αOFF-S cells in the 4wk Ctrl and 4wk IOP groups binned into 200 ms intervals during light stimulation (yellow). **(H)** Mean (left) and peak (right) light-evoked spike rates for αOFF-S cells. Error bars: ± standard error of the mean. *p < 0.05.

After 4 weeks mice were sacrificed and retinas prepared for electrophysiologic recordings. As previously described (Risner et al., 2018, 2020a, 2021, 2022; Boal et al., 2022), we targeted αRGCs for recording by identifying large cell bodies and confirmed cell types by characterizing soma size, dendritic stratification within the inner plexiform layer (Famiglietti and Kolb, 1976; Galli-Resta et al., 2000), and light-evoked responses (Figure 2). We focused analysis on two well-characterized and readily identifiable αRGC types: αON-Sustained (αON-S) and αOFF-Sustained (αOFF-S; Krieger et al., 2017). In the saline (4wk Ctrl) group we recorded 10 αON-S RGCs (7 eyes, 4 mice; 8 cells from males, 2 from females) and 10 αOFF-S RGCs (8 eyes, 7 mice; 5 male, 5 female). In the microbead group (4wk IOP) we recorded 10 αON-S RGCs (9 eyes, 5 mice; 4 male, 6 female) and 7 αOFF-S RGCs (7 eyes, 5 mice; 3 male, 4 female). Cells from mice of both sexes were evenly represented among αON-S and αOFF-S for 4wk Ctrl (*p* = 0.1596, Chi-squared test) and 4wk IOP (*p* = 0.9062, chi-squared test).

Four weeks of IOP elevation altered the resting membrane and light-evoked spiking characteristics of both RGC types. RGCs from the 4wk IOP group had a depolarized resting membrane potential (RMP) relative to controls (Figure 2C, *p* = 0.0572; αON-S + 2.25 mV, αOFF-S + 2.41 mV). Spontaneous spiking in the absence of light also appeared altered (Figure 2D), with αON-S cells trending toward greater spiking (*p* = 0.0507) and αOFF-S cells trending toward less spiking (*p* = 0.1613). The membrane voltage response to light stimulation for αON-S was significantly blunted after IOP elevation (Figures 2E,F), with cells from the 4wk IOP group exhibiting diminished mean (*p* = 0.0202) and peak (*p* = 0.0251) spike rates in response to light onset. Light-evoked spiking also appeared altered in αOFF-S cells (Figures 2G,H), although not quite as overtly as αON-S cells. Mean and peak spike rates were not significantly different between experimental groups (Figure 2H), though the histogram of mean spike rates (Figure 2G) appeared qualitatively altered by 4wk IOP, with spiking at light offset tending to be less sustained.

### 3.2. 4wk IOP RGCs are less sensitive to elevated extracellular potassium

**Figure 3:**
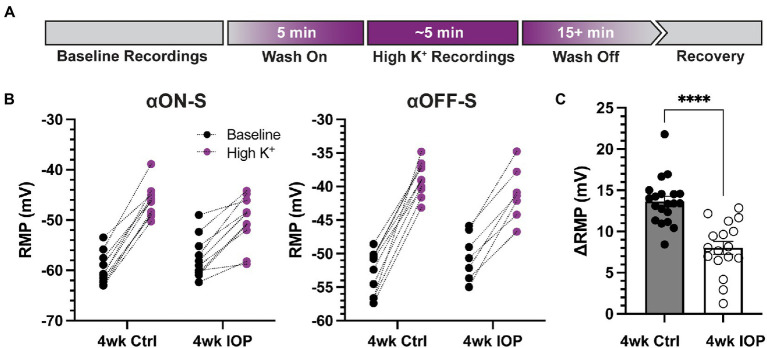
Elevated IOP reduces the influence of extracellular potassium on RGC depolarization. **(A)** Timeline illustrating the design of acutely elevated extracellular potassium (High K^+^) experiments. Following baseline recordings, extracellular medium with an extra 5 mM KCl is washed on for 5 min until membrane potentials stabilized. High K^+^ recordings are done, and then high K^+^ is washed off with regular extracellular medium until full recovery of membrane potential and spontaneous spiking, at least 15 min. **(B)** Resting membrane potentials (RMPs) for αON-S and αOFF-S cells in both experimental groups before and after high K^+^ wash on. There is a significant interaction effect between K^+^ and IOP for both αON-S (*p* = 0.0002; 2-way repeated measures ANOVA) and αOFF-S (0.0063) cells. **(C)** The change in RMP following high K^+^ wash on for each cell, separated by experimental group. Cells exposed to 4wk IOP elevation are significantly less depolarized by high K^+^ (*p* = 0.00000091, Mann–Whitney test). Error bars: ± standard error of the mean. ^****^p < 0.0001.

Since potassium homeostasis may be altered in glaucoma (Fischer et al., 2019a,b), we next sought to investigate how 4 weeks of IOP elevation alters the sensitivity of RGCs to acutely elevated extracellular potassium. As previously described (Boal et al., 2022), we performed a within-subjects experimental design with recordings before and after application of extracellular medium containing additional KCl (extra 5 mM, high K^+^, Figure 3A). For both αON-S and αOFF-S high K^+^ depolarized the RMP, regardless of experimental group (Figure 3B, *p* < 0.0001 for both cell types). However, there was a statistically significant interaction between IOP group and potassium effect on RMP for both cell types (*p* = 0.0002, αON-S; *p* = 0.0063, αOFF-S). Comparison of the high K^+^-evoked depolarization of RMP (∆RMP) between experimental groups demonstrated that 4wk IOP RGCs were significantly less depolarized by the acutely elevated potassium (Figure 3C, p < 0.0001). The sex of the mouse from which an RGC came was not significantly associated with ∆RMP (*p* = 0.1572, multiple linear regression model).

**Figure 4:**
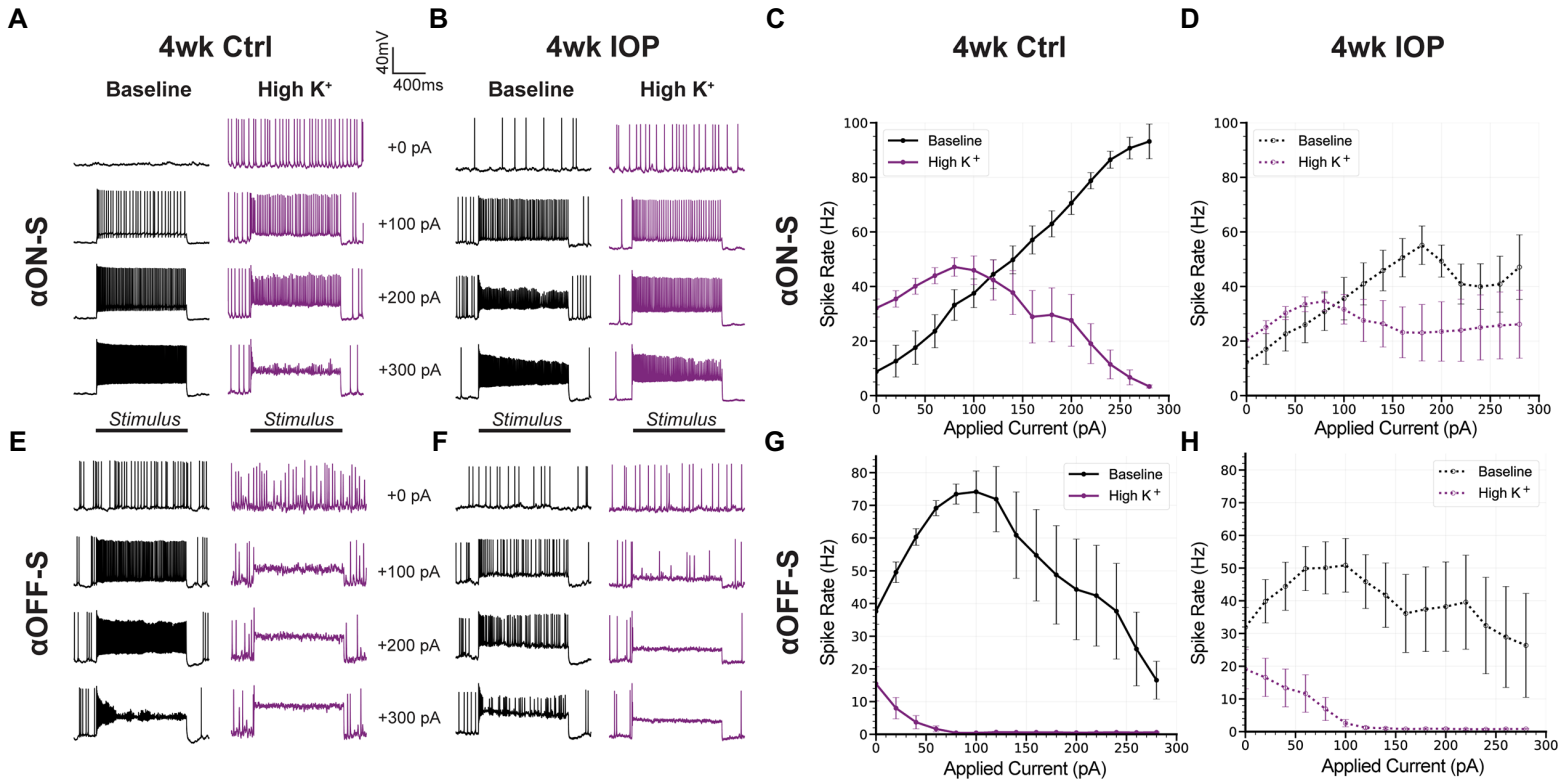
Current-evoked spiking is less depressed by high K^+^ after IOP elevation. **(A,B)** Representative current-clamp responses of αON-S cells from both experimental groups to 0, 100, 200, and 300 pA pulses, before and after washing on high K^+^. **(C,D)** The spiking responses of αON-S cells to depolarizing current pulses ranging from 0 to 300 pA, before and after high K^+^. **(E,F)** Representative current-clamp responses of αOFF-S cells from both experimental groups before and after washing on high K^+^. **(G,H)** The spiking responses of αON-S cells to depolarizing current pulses, before and after high K^+^. The difference in spike rates between baseline and high K^+^ groups for all cells is lower in the 4wk IOP group than in the 4wk Ctrl (*p* = 0.0317, unpaired *t*-test). Error bars: ± standard error of the mean.

αON-S and αOFF-S RGCs have distinct responses to depolarizing current (Twyford et al., 2014; Kameneva et al., 2016), which are in part related to their different sensitivities to extracellular potassium (Boal et al., 2022). We measured the spiking response of αRGCs to 1 s depolarizing current injections ranging from 0 to +300 pA, before and after washing on high K^+^ medium (Figure 4), to determine how 4wk IOP exposure alters these properties. In saline controls, high K^+^ appreciably altered the current-spiking relationship of both αON-S and αOFF-S cells. In baseline extracellular media control αON-S RGCs (Figures 4A,C) exhibited little spiking at low depolarizations but spike rates increased as the strength of depolarization increased. After high K^+^ wash on, spike rates were higher at small depolarizations but began to plateau and then slow as the strength of depolarization was increased. Control αOFF-S RGCs (Figures 4E,G) exhibited a different pattern of current-evoked spiking than αON-S, but were also appreciably impacted by high K^+^. In baseline media control αOFF-S cells had relatively high spike rates that initially increased with increasing stimulation, but reached a peak and began to subsequently decrease beyond about 100 pA of depolarization. High K^+^ considerably decreased spike rates, which quickly fell to 0 Hz with increasing magnitudes of depolarization. In 4wk IOP αRGCs, high K^+^ appeared to have a lesser effect on current-evoked spiking than in controls. For αON-S cells (Figures 4B,D), 4wk IOP excitability was diminished at baseline relative to controls, exhibiting less of an increase in spike rate with increasing stimulation (*p* < 0.001, simple linear regression). High K^+^ again blunted spike rates (*p* = 0.0002), though not to the same degree as in controls. 4wk IOP exposure likewise diminished αOFF-S excitability at baseline (Figures 4F,H), with peak firing rates trending lower (*p* = 0.0639, unaired t test), and lessened the impact of high K^+^ on spiking, with cells appearing to maintain the ability to fire at greater magnitudes of depolarization than in controls. Across both cell types, the absolute difference in firing rates between high K^+^ and baseline conditions was less in the 4wk IOP group than in the 4wk control group (*p* = 0.0317).

**Figure 5:**
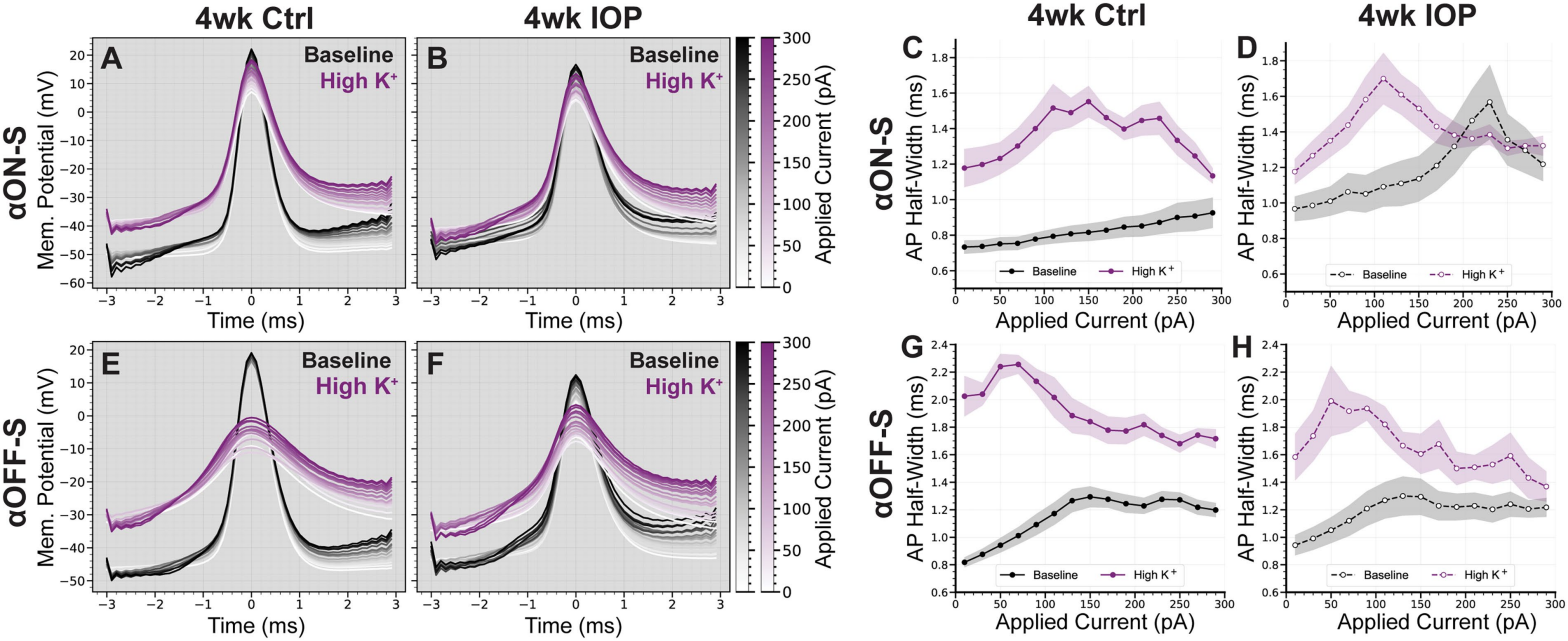
IOP elevation reduces the influence of high K^+^ on action potential shape. **(A,B)** Mean action potential (AP) shapes of αON-S cells in baseline and high K^+^ conditions at each current step, for 4wk Ctrl **(A)** and 4wk IOP **(B)** groups. **(C,D)** Action potential half-widths of αON-S cells in baseline and high K^+^ conditions at each current step, for 4wk Ctrl **(C)** and 4wk IOP **(D)** groups. **(E,F)** Mean action potential (AP) shapes of αOFF-S cells in baseline and high K^+^ conditions at each current step, for 4wk Ctrl **(E)** and 4wk IOP **(F)** groups. **(G,H)** Action potential half-widths of αOFF-S cells in baseline and high K^+^ conditions at each current step, for 4wk Ctrl **(C)** and 4wk IOP **(D)** groups. The potassium-induced widening of action potentials is lessened after 4wks IOP elevation (*p* = 0.0061, unpaired *t*-test). Shaded regions: ± standard error of the mean.

We previously found differences in excitability between αON-S and αOFF-S were reflected in the shape of their action potentials (APs), and that high K^+^ promoted rate-dependent AP widening (Boal et al., 2022). We measured AP half-widths in both experimental groups to determine if decreased potassium sensitivity in 4wk IOP RGCs was reflected at the level of AP generation (Figure 5). Control αON-S cells (Figures 5A,C) exhibited minimal AP widening with increased stimulation at baseline. High K^+^ media widened αON-S APs and increased rate-dependent widening. Control αOFF-S cells (Figures 5E,G) had a moderate degree of rate-dependent AP widening at baseline, and high K^+^ caused further widening. After 4wk IOP elevation, αON-S APs (Figures 5B,D) had slightly wider APs at baseline than controls (*p* = 0.0133, unpaired t test). However, 4wk IOP αON-S APs appeared less widened in high K^+^ medium relative to baseline. 4wk IOP αOFF-S cells (Figures 5F,H) did not have appreciably different AP shapes at baseline compared to controls (*p* = 0.6867). Further, they too had less K^+^ induced AP widening than control αOFF-S cells. In total, the mean change in AP half-width after high K^+^ application for all cells was significantly less for 4wk IOP RGCs than for controls (*p* = 0.0061).

**Figure 6:**
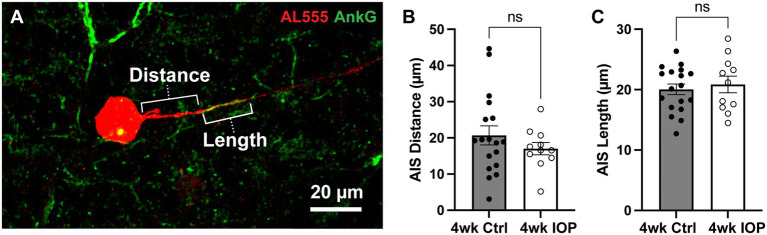
Axon initial segment dimensions are unchanged by IOP elevation. **(A)** Representative image of Alexa 555 (AL555, red) dye-filled RGC labeled for the axon initial segment (AIS) scaffolding protein ankyrin-G (AnkG, green). Annotations demonstrate the dimensions of AIS distance from soma and length which are quantified. **(B,C)** The AIS distance from the soma **(B)** and length **(C)** for all RGCs with AnkG-labeled axons. 4wk IOP does not significantly alter either of these dimensions (Distance: *p* = 0.3194, unpaired *t*-test; Length: *p* = 0.6007, unpaired *t*-test). Error bars: ± standard error of the mean.

Finally, we explored a potential mechanism for the observed differences in RGC excitability and potassium sensitivity. Scaling of the axon initial segment (AIS) is implicated in mediating intrinsic excitability of RGCs (Raghuram et al., 2019; Werginz et al., 2020; Wienbar and Schwartz, 2022). The AIS, marked by labeling for scaffolding protein ankyrin-G (AnkG), is a complex that clusters voltage-gated ion channels in the proximal portion of the axon and serves as the site of AP generation (Zhou et al., 1998; Gasser et al., 2012; Huang and Rasband, 2018; Leterrier, 2018). The dimensions of the AIS are plastic and can change in response to stimuli, such as chronically elevated extracellular potassium (Grubb and Burrone, 2010) and sustained sensory input (Jamann et al., 2021), in order to modulate neuronal excitability. Because changes to the AIS have been implicated in neurodegenerative disease (Sun et al., 2014; Marin et al., 2016; Hatch et al., 2017), we investigated whether altered AIS dimensions were associated with the decreased RGC potassium sensitivity in our microbead model. We labeled filled RGCs for AnkG and measured the AIS distance from the soma and length (Figure 6A) for each RGC axon. There were 11 4wk IOP cells (5 αON-S and 6 αOFF-S) and 18 4wk Ctrl cells (9 αON-S and 9 αOFF-S) with identifiable axon initial segments. The AIS distance (Figure 6B) and length (Figure 6C) from 4wk IOP RGCs was not statistically different than those of control RGCs.

## 4. Discussion

### 4.1. Blunted RGC excitability occurs alongside a reduced sensitivity to high K^+^ conditions

The data presented here support evidence of RGC excitability changes with prolonged exposure to elevated IOP and offer insight into how RGCs respond to the acute stress of elevated extracellular potassium. We hypothesized intrinsic differences in K^+^ sensitivity between αON-S and αOFF-S RGCs may drive a preferential susceptibility to elevated IOP-induced degeneration. In the present study we did not evaluate the degree of RGC death by counting somas in the retina or axons in the optic nerve. Previous work in the same model has established at the four-week timepoint there is some degeneration of axons but minimal loss of RGC somas in the retina (Ward et al., 2014; Bond et al., 2016; Risner et al., 2021, 2022). As in previous experiments at the four-week time point (Risner et al., 2021, 2022) we observed reduced light-and current-evoked RGC spiking (Figures 2, 4). Though the excitabilities of both αRGC types appear altered in the 4wk IOP group relative to controls, there appears to be a marginally larger effect size on the αON-S cells. These findings could represent a preferential susceptibility to IOP-related stress; however, excitability changes may also be an adaptive response.

We challenged RCGs with acutely elevated extracellular K^+^ to determine how sensitivity to ionic stress changes with prolonged IOP elevation and how this impacts excitability. As expected, high K^+^ media depolarized RGC membranes for both cell types and both experimental groups (Figure 3). Remarkably, 4wk IOP elevation significantly diminished this effect, suggesting that there is decreased RGC sensitivity to acute ionic stress. We examined the impact of potassium on RGC excitability to determine if this difference was related to intrinsic changes, such as altered axonal K^+^ ion channel expression or function (Figure 4). Stepwise application of depolarizing currents reflected previously determined cell type (Twyford et al., 2014; Kameneva et al., 2016; Yang et al., 2018; Boal et al., 2022) and IOP dependent (Risner et al., 2022) differences in RGC excitability, and high K^+^ conditions significantly impacted spiking. Strikingly, RGCs in the 4wk IOP group were less impacted by high K^+^, maintaining sustained spiking at greater magnitudes of depolarizing current before reaching the threshold for depolarization block. These findings support the notion that decreased RGC excitability and altered K^+^ sensitivity are related to RGC-intrinsic changes.

To further probe these effects, we measured AP half-width during evoked spiking (Figure 5). Differences in this measure may reflect changes to the mechanisms of AP generation, as AP shape is impacted by K+ currents (Geiger and Jonas, 2000; Kole et al., 2007; Kuznetsov et al., 2012; Gonzalez Sabater et al., 2021; Alexander et al., 2022). Again, there was a dramatic difference in the effect of K^+^ between the 4wk IOP and control groups: for both αON-S and αOFF-S, APs were less widened by high K^+^. This further supports the hypothesis that elevated IOP is affecting RGC-intrinsic excitability and suggests that there may be altered structure or function of voltage gated K^+^ channels. Interestingly, however, there was cell type specificity in how AP widths differed with IOP exposure and K^+^ conditions. αON-S cells exhibited a widening of APs following 4wk IOP elevation, even in baseline, normal K^+^ conditions. On the contrary, αOFF-S AP widths were similar for both the 4wk IOP and the control groups under normal K^+^. Both cell types had less change in AP width following high K^+^ wash on, but this difference was driven largely by the IOP-induced baseline shift for the αON-S RGCs. This is perhaps a function of cell type-specific responses to stress, paralleling the differences seen in Figure 2. αON-S RGCs had significantly diminished light-evoked spiking, while αOFF-S light spiking was mostly preserved. It remains to be determined whether these changes prove to be protective or maladaptive for the RGCs with continued stress.

### 4.2. Retinal ganglion cell adaptation to prolonged stress

The significant differences in the impact of high K^+^ conditions on RGC responses are suggestive of an adaptive process, whereby RGCs alter their physiology to preserve function and/or mitigate further degenerative stress. Hyperexcitability at 2 weeks following IOP elevation is driven by axogenic processes (Risner et al., 2018, 2020b), and these studies further support evidence of axonal changes at 4 weeks. RGC axonal excitability and AP generation is dependent upon and shaped by the AIS, a dynamic structure, thus we focused our mechanistic exploration on alterations to the AIS dimensions. We hypothesized that, similar to *in vitro* chronic depolarization (Grubb and Burrone, 2010), prolonged glaucomatous stress from K^+^ dysregulation and early hyperexcitability would lead to a distal shift in the AIS away from the soma. Yet, the results shown in Figure 6 do not demonstrate any differences in AIS dimensions between 4wk IOP cells and controls. Though this interpretation is limited by sample size and the lack of topographic location of cells, since AIS dimensions scale with retinal topography (Raghuram et al., 2019), this finding indicates that our observed differences in excitability and K^+^ sensitivity are likely not solely reflective of AIS structural plasticity.

Rather, changes in voltage-gated ion channel and interacting protein expression, alongside larger scale alterations in glial regulation of the extracellular milieu (Nwaobi et al., 2016; Murphy-Royal et al., 2017; Fischer et al., 2019b; Theparambil et al., 2020; Boal et al., 2021), may underly a multifactorial adaptive process to minimize metabolic and excitotoxic stress. Retinal regulation of extracellular K^+^ is largely accomplished by Müller glia (Newman et al., 1984; Karwoski et al., 1989; Kofuji and Newman, 2004), which undergo reactive changes in glaucoma and exhibit physiologic deficits in K^+^ buffering capacity (Bolz et al., 2008; Fischer et al., 2019b). RGC hyperexcitability driving increased K^+^ flux may compound with impaired glial buffering capacity, amplifying axonal stress. Furthermore, depressed excitability may reflect interactions between dysregulated potassium and alterations in other ions, such as calcium, which modulates neuronal excitability and can contribute to cell death (Jones and Smith, 2016; Segal, 2018). Investigation of changes to expression and function of calcium-activated potassium channels in this model may further elucidate ion-mediated mechanisms of glaucomatous degeneration (Stirling and Stys, 2010; Crish and Calkins, 2011; Van Hook et al., 2019).

Glaucoma is a chronic and insidious disease, where the interaction between vulnerable RGCs and physiologic stress can lead to dysfunction and cell death over the course of many years. It often takes a significant degree of axon degeneration for many patients notice the resultant vision changes (Hu et al., 2014). While this emphasizes the importance of early diagnosis, it also suggests a resilience of visual function in the face of prolonged stress. Discoveries in animal models of glaucoma have illuminated the variety of adaptive responses that RGCs undergo in the face of oxidative, metabolic, and inflammatory challenges (Calkins, 2021). The experiments presented here explore an important facet of RGC adaptation, giving insight into the modulation of RGC excitability, and lay the groundwork for mechanistic investigation into potential diagnostic and therapeutic targets in early glaucomatous neurodegeneration.

## Data availability statement

The raw data supporting the conclusions of this article will be made available by the authors, without undue reservation.

## Ethics statement

The animal study was reviewed and approved by Vanderbilt University Medical Center Institutional Animal Care and Use Committee.

## Author contributions

AB, MR, and DC designed research. AB and NM performed research. AB, NM, and JH analyzed data. AB and DC wrote the paper. All authors contributed to the article and approved the submitted version.

## Funding

This work was supported by the Potocsnak Family Vision Research Center, a departmental unrestricted award by the Research to Prevent Blindness Inc., Research to Prevent Blindness Inc. Stein Innovation Award to DC, and National Institutes of Health grants EY017427, EY024997, and EY008126 to DC. AB was supported in part by NEI and NIGMS of the National Institutes of Health under award numbers 1F30EY033627-01A1 and T32GM007347. MR was supported in part by BrightFocus Foundation Award G2022011S. Imaging supported through the Vanderbilt University Medical Center Cell Imaging Shared Resource core facility and NIH grants CA68485, DK20593, DK58404, and DK59637.

## Conflict of interest

The authors declare that the research was conducted in the absence of any commercial or financial relationships that could be construed as a potential conflict of interest.

## Publisher’s note

All claims expressed in this article are solely those of the authors and do not necessarily represent those of their affiliated organizations, or those of the publisher, the editors and the reviewers. Any product that may be evaluated in this article, or claim that may be made by its manufacturer, is not guaranteed or endorsed by the publisher.
